# Prevalence of obesity and its association with cardiometabolic risk factors, heart failure phenotype and mortality among patients hospitalized for heart failure in Egypt

**DOI:** 10.1186/s43044-021-00232-y

**Published:** 2022-01-03

**Authors:** Ahmed Hassanin, Mahmoud Hassanein, Gregg M. Lanier, Mohamed Sadaka, Mohamed Rifaat, Mohamed Sanhoury

**Affiliations:** 1 Westchester Medical Center/New York Medical College, Valhalla, USA; 2 Alexandria University, Alexandria, Egypt; 3 Ministry of Health, Cairo, Egypt

**Keywords:** Egypt, Obesity, Heart failure, Co-morbidities, Mortality

## Abstract

**Background:**

Obesity is an established risk factor for cardiometabolic disease and heart failure (HF). Nevertheless, the relationship between obesity and HF mortality remains controversial.

**Results:**

The goal of this study was to describe the prevalence of obesity in patients hospitalized for HF in Egypt and investigate the relationship of obesity to cardiometabolic risk factors, HF phenotype and mortality. Between 2011 and 2014, 1661 patients hospitalized for HF across Egypt were enrolled as part of the European Society of Cardiology HF Long-term Registry. Obese patients, defined by a BMI ≥ 30 kg/m^2^, were compared to non-obese patients. Factors associated with mortality on univariate analysis were entered into a logistic regression model to identify whether obesity was an independent predictor of mortality during hospitalization and at one-year follow-up. The prevalence of obesity was 46.5% and was higher in females compared to males. Obese as compared to non-obese patients had a higher prevalence of diabetes mellitus (47.0% vs 40.2%, *p* = 0.031), hypertension (51.3% vs 33.0%, *p* < 0.001) and history of myocardial infarction (69.2% vs 62.8% *p* = 0.005). Obese patients as compared to non-obese patient were more likely to have acute coronary syndrome on admission (24.8% vs 14.2%, *p* <  < 0.001). The dominant HF phenotype in obese and non-obese patients was HF with reduced ejection fraction (EF); however, obese patients as compared to non-obese patient had higher prevalence of HF with preserved EF (22.3% vs 12.4%, *p* < 0.001). Multivariable analysis demonstrated that obesity was associated with an independent survival benefit during hospitalization, (OR for mortality 0.52 [95% CI 0.29–0.92]). Every point increase in BMI was associated with an OR = 0.93 [95% CI 0.89–0.98] for mortality during hospitalization. The survival benefit was not maintained at one-year follow-up.

**Conclusions:**

Obesity was highly prevalent among the study cohort and was associated with higher prevalence of cardiometabolic risk factors as compared to non-obese patients. Obesity was associated with an independent “protective effect” from in-hospital mortality but was not a predictor of mortality at 1-year follow-up.

## Background

Heart failure (HF) is a major global health challenge that is increasing in scope and severity in many countries [1–5]. Despite recent therapeutic advances, HF morbidity and mortality remain high. Similarly, obesity, defined as body mass index (BMI) over 25 kg/m^2^, is a global public health problem with over 2.2 billion people meeting the definition of overweight or obese in 2015 [6]. Obesity is a major risk factor for ischemic heart disease, which in turn is the most common etiology for HF with reduced ejection fraction (HFrEF). Additionally, obesity is strongly associated with left ventricular diastolic dysfunction and HF with preserved ejection fraction (HFpEF).

Accordingly, primary prevention of cardiometabolic syndrome and mitigation of established cardiovascular risk factors, which encompasses obesity, is an important component of standard HF management. However, the relationship between obesity and HF mortality has been controversial, with several large studies suggesting a “protective effect” of obesity in HF patients [7–9]. The data on this relationship has been largely confined to patients’ cohorts from western countries. In this analysis of a large cohort of patients admitted with decompensated HF in Egypt, we investigated the prevalence of obesity and its association with cardiometabolic risk factors, HF phenotype and mortality.

## Methods

This study utilizes data from the Egyptian cohort of European Society of Cardiology (ESC) Heart Failure Long-term (ESC-HF-LT) Registry, which has been reported in detail elsewhere [10, 11]. Briefly, this is a multi-center, observational registry of patients from European and Mediterranean countries which are members of the ESC. Twenty medical centers across Egypt participated in this registry. The enrolling centers were chosen to truly reflect the heterogeneity and the Egyptian health care system and patient population. Between April 2011 and September 2014, 1661 patients hospitalized for decompensated HF in Egypt were enrolled, including patients with pre-existing or new diagnosis of HF. The diagnosis of HF was determined according to the clinical judgement of participating centers’ treating cardiologist. There were no exclusion criteria for this registry; however, all patients had to be adults in order to consent for participation in this study. The registry was approved by the institutional review board of the enrolling centers and in compliance with the Declaration of Helsinki.

Patients were classified into 5 categories according to body BMI: underweight (BMI < 18.5 kg/m^2^), normal body weight (BMI: 18.5–24.9 kg/m^2^), overweight (BMI: 25–29.9 kg/m^2^), obese (BMI: 30–39.9 kg/m^2^) and morbidly obese (BMI ≥ 40 kg/m^2^). For the purpose of this analysis, obese and morbidly obese patients were categorized as “obese”, while underweight, lean and overweight were categorized as non-obese. Chronic kidney disease (CKD) was defined as a serum creatinine > 1.5 mg/dl and anemia was defined as a hemoglobin ≤ 12 mg/dl. Continuous variables were expressed as mean with standard deviation, while categorical variables were represented as frequencies and rates. For categorical variables, the difference between obese and non-obese was analyzed using chi-square test. Unpaired t-test was used to compare continuous variables between obesity classes. Variables with *P* values < 0.05 on univariate analysis were introduced in the multivariable logistic regression model. Odds ratio (OR) was calculated for factors that were found to be independently associated with mortality. All tests were two sided and a *P* value of < 0.05 was considered significant. All analyses were done using SPSS for windows, version 21 (SPSS, Inc., Chicago, IL, USA).

## Results

Between April 2011 and September 2014, 1661 patients hospitalized with HF were recruited by the enrolling centers across Egypt. A total of 53 patients were excluded due to missing BMI data, leaving 1608 patients who were eligible for analysis. Two thirds of the participants were males and one third were females.

**Fig. 1:**
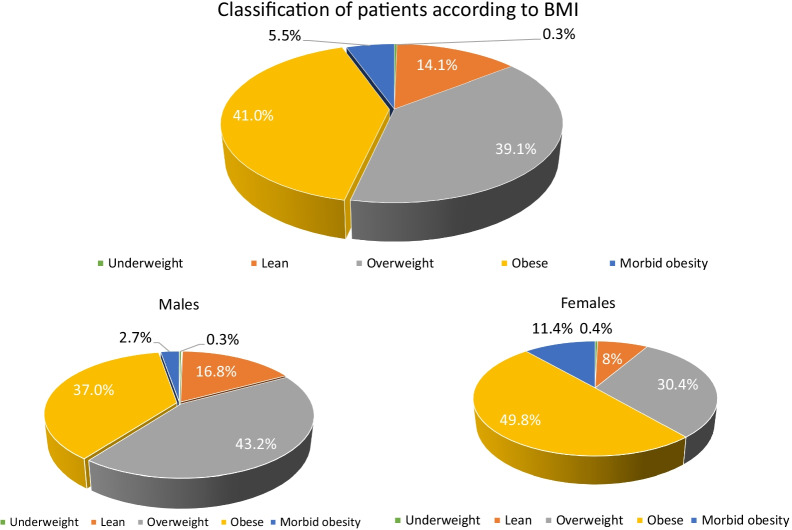
Prevalence of obesity among hospitalized heart failure patients. BMI: Body mass index

Figure 1 demonstrates that the nearly half of the patients (46.5%) were either obese or morbidly obese. Females were more likely to be either obese or morbidly obese as compared to males (61.2% vs 39.7%). The differences in baseline characteristics between obese and non-obese patients are depicted in Table 1. Obese patients had a higher systolic blood pressure (SBP) on presentation (142.4 ± 33.6 vs 123.8 ± 27.5, *P* < 0.001) and higher heart rate (106.9 ± 23.6 vs 99.8 ± 19.6, *P* < 0.001) and were more likely to have acute coronary syndrome on admission (24.8% vs 14.2%, *P* <  < 0.001). Obese patient had greater prevalence of diabetes mellitus (47.0% vs 40.2%, *P* = 0.031), hypertension (51.3% vs 33.0%, *P* < 0.001), and history of myocardial infarction (69.2% vs 62.8%. *P* = 0.005). Non-obese patients were more likely to be smokers (68.5% vs 49.9%, *P* < 0.001) and have history of hepatic dysfunction (12.7% vs 5.9%, *P* < 0.001). Although the dominant phenotype of HF in obese and non-obese patients was HFrEF, the prevalence of both HFpEF and HF with mid-range ejection fraction (HFmrEF) were significantly higher in obese patients, 22.3% versus 12.4%, *P* < 0.001 and 19.1% versus 15.1%, *P* < 0.001, respectively.

**Table 1:** Baseline characteristics among obese and non-obese patients with heart failure

		Obese (*n* = 750)	Non-obese (*n* = 853)	*P* value
Age (years)		60.59 ± 10.7	59.58 ± 13.9	0.117
Male		58.0 (435)	75.9 (647)	< 0.001
*Presentation*				
SBP (mmHg)		142.41 ± 33.6	123.82 ± 27.5	< 0.001
HR (beat/min)		106.96 ± 23.6	99.89 ± 19.6	< 0.001
NYHA class	III	59.3 (416)	61.2 (417)	0.48
	IV	40.7 (286)	38.8 (264)	
Hospital presentation	ACS/HF	24.8 (188)	14.2 (106)	< 0.001
	Cardiogenic shock	2.1(16)	5.6 (42)	
	Decompensated HF	46.1 (345)	62.9 (470)	
	Hypertensive HF	8 (59)	2 (15)	
	Pulmonary edema	14.8 (111)	9.9 (74)	
	Right HF	4.1 (30)	5.4 (40)	
*HF etiology and cardiac evaluation*				
Primary etiology	DCM	14.1 (104)	19.7 (144)	< 0.001
	HTN	6.3 (46)	2.2 (16)	
	Ischemic	68.6 (505)	62.1 (454)	
	Valve disease	7.3 (54)	10 (73)	
	Other	3.7 (27)	6 (44)	
EF (%)	40.89 ± 12.6		36.7 ± 11.7	< 0.001
EF	< 40	58.6 (346)	72.5 (375)	< 0.001
	40–49	19.1 (113)	15.1 (78)	
	≥ 50	22.3 (132)	12.4 (67)	
*Comorbidities*				
History of MI	69.2 (519)		62.8 (470)	0.005
Smoking	49.9 (750)		68.5 (749)	< 0.001
Diabetes	47.0 (352)		40.2 (312)	0.031
HTN	51.3 (385)		33.0 (247)	< 0.001
AF	27.4 (196)		25.4 (179)	0.22
CKD	17.2 (129)		16.2 (121)	0.628
COPD	12.4 (93)		14.6 (109)	0.227
Stroke	6.7 (50)		8.7 (65)	0.147
PVD	5.3 (40)		4.8 (36)	0.724
Hepatic dysfunction	5.9 (44)		12.7 (95)	< 0.001
Anemia	70.8 (402)		62.5 (408)	0.001

Values are n (%) or mean (± SD)

ACS: acute coronary syndrome; AF: Atrial fibrillation; CKD: chronic kidney disease; COPD: chronic obstructive pulmonary disease; EF: Ejection fraction; HR: Heart rate; HF: heart failure, HTN: Hypertension; MI: Myocardial infarction; NYHA: New York Heart Association; PVD: Peripheral vascular disease; SBP: Systolic blood pressure

Table 2 illustrates differences between obese HF patients according to sex. The prevalence of obesity was significantly higher in females compared to males (61.3% vs 39.7%, *P* < 0.001). As compared to obese men, obese women were older (61.6 ± 10.9 vs 59.8 ± 10.5, *P* = 0.12) and had a higher prevalence of hypertension (57.5% vs 52.1%, *P* = 0.005), diabetes (52.1% vs 43.3%, *P* = 0.06) and anemia (87.2% vs 58.4%, *P* < 0.001). Obese women also presented with a higher left ventricular (LV) ejection fraction (43.5 ± 13.4 vs 38.8 ± 11.6, *P* < 0.001). HFpEF (LV ejection fraction > 50%) was present in 31.9% of obese women versus 15.1% of obese men (*P* < 0.001). Atrial fibrillation was significantly more prevalent in obese women (34.4% vs 22.5% *P* < 0.001).

**Table 2:** Baseline characteristics among obese men and obese women with heart failure

		Obese Men (*n* = 435)		Obese Women (*n* = 315)	*P* value
Age (years)		59.8 ± 10.5		61.6 ± 10.9	0.117
*Presentation*					
SBP (mmHg)		139.73 ± 31.3		146.1 ± 36.2	0.01
HR (beat/min)		105.64 ± 21.7		108.78 ± 25.9	0.073
NYHA class	III	60.7 (246)		57.4 (171)	0.39
	IV	39.3 (149)		42.6 (127)	
Hospital presentation	ACS/HF	28.3 (123)		20.1 (63)	0.21
	Cardiogenic shock	1.1 (43)		3.5 (11)	
	Decompensated HF	47.6 (207)		43.9 (138)	
	Hypertensive HF	6 (26)		10.8 (34)	
	Pulmonary edema	14.5 (63)		15.3 (48)	
	Right HF	2.5 (11)		6.4 (20)	
*Cardiac evaluation*					
EF (%)	38.8 ± 11.6			43.5 ± 13.4	< 0.001
EF	< 40	64.8 (219)		50.4 (128)	< 0.001
	40–49	20.1 (68)		17.7 (43)	
	≥ 50	15.1 (51)		31.9 (81)	
Primary etiology	DCM		14.8 (63)	13.2 (41)	0.46
	HTN		4.9 (21)	8 (25)	
	Ischemic		69.6 (296)	67.2 (209)	
	Valve disease		6.8 (29)	8 (25)	
	Other		3.8 (16)	3.5 (11)	
*Comorbidities*					
History of MI	74.9 (326)			61.3 (193)	< 0.001
Smoking	80 (348)			8.3 (26)	< 0.001
Diabetes	43.3 (187)			52.1 (163)	0.006
HTN	52.1 (204)			57.5 (181)	0.005
AF	22.5 (95)			34.4 (101)	0.001
CKD	15.2 (66)			20.0 (63)	0.09
COPD	16.3 (71)			7.0 (22)	0.001
Stroke	5.5 (24)			8.2 (26)	0.141
PVD	5.7 (25)			4.8 (15)	0.62
Hepatic dysfunction	6.7 (29)			4.8 (15)	< 0.001
Anemia	58.4 (198)			87.2 (224)	< 0.001

Values are n (%) or mean (± SD)

ACS: acute coronary syndrome; AF: Atrial fibrillation; CKD: chronic kidney disease; COPD: chronic obstructive pulmonary disease; EF: Ejection fraction; HR: Heart rate; HF: heart failure, HTN: Hypertension; MI: Myocardial infarction; NYHA: New York Heart Association; PVD: Peripheral vascular disease; SBP: Systolic blood pressure

Table 3 demonstrates that in-hospital mortality was higher in non-obese patients compared to obese patients (7.6% vs 2.9%, *P* < 0.001), particularly in non-obese men. This difference in mortality between non-obese and obese patients was not seen at 1 year follow-up (28.0% vs 25.7%, *P* = 0.41).

**Table 3:** In-hospital and one-year mortality for the entire cohort and among obese and non-obese men and women with heart failure

Mortality	Entire cohort			Men			Women		
	Obese	Non-obese	*P* value	Obese	Non-obese	*P* value	Obese	Non-obese	*P* value
In-hospital	2.9 (21)	7.6 (56)	< 0.001	1.6 (7)	7.3 (42)	< 0.001	4.6 (14)	8.8 (14)	0.06
1 year	25.7 (152)	28.0 (149)	0.41	24.2 (83)	26.9 (115)	0.408	27.7 (69)	32.1 (34)	0.24

Values are n (%) or mean (± SD)

Table 4 demonstrates the factors associated with survival at hospital discharge. On univariate analysis, obesity (OR 2.85, 95% CI [1.68–4.7]), a higher admission SBP (1.58, 95% CI [1.43–1.79], for every 10 points increase), and history of myocardial infarction (OR 1.75, 95% CI [1.11–2.74]) were associated with improved survival; while CKD (OR 0.31, 95% CI [0.19–0.50]) was associated with lower survival. Multivariable analysis, adjusted for age, sex, obesity, SBP, and history of myocardial infarction, confirmed that obesity was associated with an independent survival benefit during the in-hospital course (OR 1.92, 95% CI [1.10–3.44]). Every point increase in BMI was associated with an OR of 0.93 (95% CI 0.89–0.98) for mortality. At one-year follow-up, obesity was not associated with mortality on univariate or multivariable analysis. Age, LV ejection fraction, CKD and anemia were associated with survival on both univariate and multivariable analysis (Table 5).

**Table 4:** Factors associated with survival at hospital discharge

Clinical variable	Univariate analysis			Multivariable analysis		
	OR	95% CI	*P* value	OR	95% CI CI	*P* value
Obesity	2.85	1.68–4.7	< 0.0001	1.92	1.1–3.44	0.026
SBP ^#^	1.58	1.43–1.79	< 0.0001	1.67	1.35–1.85	> 0.001
History of MI	1.75	1.11–2.74	0.01	–-	–	–
CKD	0.312	0.19–0.50	< 0.001	0.32	0.15–0.66	0.002

CKD: chronic kidney disease; SBP: Systolic blood pressure; MI: Myocardial infarction

^#^SBP—for every 10 point increase

**Table 5:** Factors associated with survival at one-year follow-up

Clinical variable	Univariate analysis			Multivariable analysis		
	OR	95% CI	*P* value	OR	95% CI	*P* value
Obesity*	–	–	–	–	–	–
Age^	1.72	1.56–1.96	< 0.0001	1.47	1.25–1.56	< 0.0001
EF ~	1.32	1.22–1.40	< 0.0001	1.52	1.37–1.69	< 0.0001
CKD	0.49	0.37–0.62	< 0.0001	0.55	0.31–0.94	0.03
Anemia	0.625	0.46–0.84	< 0.0001	0.59	0.38–0.9	0.02

EF: Ejection fraction; CKD: chronic kidney disease

*Obesity was not association with survival at one-year follow-up

^Age for every 10 years below 75

~ Ejection Fraction—for every 5% increase above 25%

## Discussion

The main findings of this study:

1. Obesity was highly prevalent among patients admitted for decompensated HF in Egypt: 39.7% of males and 61.2% of females were either obese or morbidly obese.
2. Among obese patients, hypertension, diabetes, and anemia were more common as compared to non-obese patients.
3. Among obese patients, HFpEF prevalence was nearly double that of non-obese patients
4. Obesity was associated with a “protective effect” from inhospital mortality, but this effect was absent at 1-year follow-up.

### Prevalence of obesity in Egypt

The Global Burden of Disease 2015 Obesity Collaborators study reported that among the 20 most populous countries, the highest level of age-standardized adult obesity was observed in Egypt (35.3%, 95% CI [33.6 to 37.1]) [6]. The Demographic and Health Survey (DHS) conducted in Egypt in 2015 reported that prevalence of obesity among adult women was double that of men, 50.3% versus 26.4% [12]. The prevalence of obesity in women was higher than men across all age brackets, with the peak prevalence of obesity in women observed in both sexes in the 55–59 years age group. Obesity in Egyptian women correlated with age, from less than 10% among adolescent women to over 65% in women aged 55–59 years. In large survey conducted in Cairo in 2007, Egypt’s largest city, obesity was prevalent across all strata of socioeconomic classes [13]. Further analysis of the DHS 2008 data demonstrated that in women with the low educational attainment but high economic means had the highest prevalence of obesity [14].

In the ESC-HF-LT Registry, the median BMI of patients hospitalized for HF in Egypt was 29.4 kg/m^2^ (IQR:26.5–33.2) as compared to 27.7 kg/m^2^(IQR: 24.2–31.2) [9] in their European counterparts, but was similar to that reported in the HEARTS registry carried out in Saudi Arabia (29.2 kg/m2 ± 6.7) [13]. The obesity epidemic in Egypt is reflective of a wider regional problem seen in the World Health Organization (WHO) Eastern Mediterranean Region, in which half of all women and more than two in five men were overweight or obese [15].

Obesity, cardiometabolic disease risk factors and cardiovascular disease Diabetes mellitus: Obesity was associated with significantly higher prevalence of diabetes mellitus type 2 in this study. Diabetes was more prevalent in obese women versus obese men (52.1% vs 43.3%, *P* = 0.006). Egypt is among the top 10 countries in the world with the number of adults with diabetes in 2013, with a prevalence rate of 15.5% [16]. Obesity, especially visceral adiposity, and physical inactivity are major risk factors for diabetes in Egypt. It is hypothesized that adipose tissue and insulin resistance may produce a systemic proinflammatory state modulated through cytokines production that leads to myocyte remodeling and the development of HFpEF [17, 18].

### Hypertension

The prevalence of hypertension in the Egyptian cohort was 43.5% as compared to 64.5% in the European cohort of the ESC-HF-LT registry [10]. Prevalence of hypertension was higher in obese patients regardless of sex. Obese women had the highest prevalence of hypertension (57.5%). The reported prevalence of hypertension (defined as a blood pressure > 140/90) in the Egypt STEPwise survey conducted in 2017 was 29.9% for adult men and 29.2% for women [19], in comparison to the reported prevalence in the National Hypertension Project (NHP), conducted 15 years earlier, of 25.7% in men and 26.9% in women. The NHP found that 50% of the surveyed individuals had central obesity. Increasing adiposity was associated with enhanced renin angiotensin aldosterone system stimulation and sympathetic system over activation which cause increased arterial stiffness, hypertension and cardiac diastolic dysfunction [20].

### Anemia

Anemia was highly prevalent in our heart failure population, ironically more in the obese than non-obese patients. In a meta-analysis by Groenveld et al. of 34 studies, comprising 153,180 patients with HF, anemia was associated with an increased risk of mortality in both HFrEF and HFpEF [21]. Anemia can occur in heart failure with or without chronic kidney disease and is likely to be due to increased production of tumor necrosis factor-alfa and interleukin-6, which in turn can cause reduced erythropoietin secretion. The anemia can cause progressive worsening of cardiac function by increasing cardiac demand and LV hypertrophy, which in turn can lead to myocyte apoptosis.

### Ischemic heart disease

Ischemia was the dominant underlying etiology of HF in the majority of patients in this registry and it was more frequent in obese patients. Moreover, acute coronary syndrome on presentation was more common in obese patients. In a large meta-analysis of 302,000 persons, overweight and obesity were independently associated with developing coronary artery disease, RR to 1.17 (1.11–1.23), independent of hypertension and dyslipidemia [22].

### Atrial fibrillation

Prevalence of atrial fibrillation (AF) in the Egyptian cohort was significantly lower than other European countries participating in the ESC-HF-LT registry (24.3% vs 48.4%, *P* < 0.0001). This difference can be explained by the younger age of the Egyptian HF patients as compared to their European counter parts. However, when we look at the gender differences in AF in the Egyptian cohort, women had more frequent AF compared to men (34.4% vs. 22.5%, *P* < 0.001). AF tended to be more frequent in the obese patients in this study (27.4 vs 25.4%) but the difference did not reach statistical significance. Several studies have demonstrated that obese patients have a 1.52 times higher risk for the development of AF compared to the normal weight population [23], and that for each point increase in BMI the frequency of new onset AF rises by 4% [24]. This relationship did not vary by age, sex, or systolic blood pressure.

### Obesity and heart failure

#### Relation of obesity to heart failure phenotype and gender

The Framingham study investigated the risk of developing HF in obese patients [25], the study found that obese subjects had double the risk for developing HF as compared to subjects with normal BMI. For every 1 kg/m^2^ increase in BMI the, risk of HF increased by 7% in women and 5% in men. Recent studies have shown significant association between BMI and LV diastolic function, which represents the cornerstone of HFpEF [26]. Analysis of the Irbesartan in Heart Failure with Preserved Ejection Fraction trial pointed out that more two thirds of patients with HFpEF were overweight or obese [27]. The Women’s Health Study demonstrated that obesity was associated with a population attributable risk of developing HFpEF that was more than 3 times higher than that of HFrEF [28]. HFrEF was the dominant phenotype of HF in this study regardless of the BMI. However, obese patients were more likely to have a preserved LV ejection fraction than non-obese patients (22.3% vs 12.4%, *P* < 0.001). We found that 31.9% of obese women had HFpEF versus 15.1% of obese men (*P* < 0.001). In patients with HFpEF, 68% were classified as obese.

The mechanism of developing HF in obese patients is thought to be mediated by increased signaling through the leptin receptor, which can promote activation of both the sympathetic nervous system and the renin-angiotensin aldosterone system [29]. Additionally, adipocytes are though to cause overactivity of vasoactive peptides such as Neprilysin [29]. This cascade of interactions leads to plasma volume expansion and adverse myocyte remodeling.

#### The obesity paradox

The results of this analysis demonstrated an in-hospital survival benefit of obesity in the entire cohort of heart failure patients. However, there was no survival benefit of obesity at one-year follow-up in either sex. Even though increased BMI and obesity put patients at risk of developing cardiovascular disease (CVD), several studies have suggested that there may be an inverse relationship between obesity and the prognosis of HF—a phenomena known as the obesity paradox.

In the meta-analysis by Oreopoulos et al. of 9 observational studies including over 28,000 HF patients with a mean follow-up of 2.7 years, obesity (adjusted HR 0.88, CI [0.83–0.93]) and overweight (adjusted HR 0.93, CI [0.89–0.97]) were associated with lower mortality [30]. In contrast, a recent meta-analysis by Marcks et al. of 5,819 individual patient level data pooled from several cohorts with a mean follow-up of 5 years, showed that the “protective effect” of obesity in HF was only seen in patients older than 75 years or having at least one relevant co-morbidity, and not in younger patients with HF only [7]. Most studies that addressed the obesity paradox assessed BMI at the time of diagnosis with HF; however, in the analysis by Khalid et al. of a community-based cohort study of over 15,000 patients, BMI was evaluated at least 6 months prior to the incident date of developing HF [9], the study’s main conclusion was that patients who are overweight or obese before the diagnosis of HF had a better prognosis after they develop HF when compared with patients with normal weight. In an analysis of 3,310 patients with HFpEF where the mean follow-up was 3.4 years, abdominal obesity was associated with an approximate 50% increase in mortality after multivariable adjustment [31], and thus implying that visceral obesity pathophysiology may differ in HF patients with preserved ejection fraction as compared to those with reduced ejection fraction.

The impact of obesity on HF should be interpreted in light of a patient’s cardiorespiratory fitness (CRF). In a cohort of 774 HF patients all of whom underwent cardiorespiratory fitness testing [32], the obesity paradox was noted in patients with low exercise capacity group but not in patients who could achieve more than 4 metabolic equivalents. The study indicated that the obesity paradox may only be applicable in HF patients with low CRF.

Multiple mechanisms have been proposed as an explanation for the obesity paradox. Obese patients may present at an earlier stage of disease as a consequence of more prominent symptoms of dyspnea associated with surplus body weight. This represents a form of lead time bias. The association between lower BMI and worse outcome may be due to advanced stage of HF and co-morbidities. Non-obese patients may lack metabolic reserve and suffer from “cardiac cachexia,” which is an established poor prognostic marker in HF. Another hypothesis is that obese patients tend to tolerate higher doses of cardioprotective HF drugs as they usually maintain a higher blood pressure. On a molecular level, obese patients have higher levels of anti-inflammatory adipokines that may neutralize circulating inflammatory endotoxins which characterizes advanced HF [33].

Despite the results of this analysis and the studies reviewed in this discussion, weight loss may be desirable in cases of HF patients with morbid obesity as it may result in improvement in exercise capacity and symptoms. The ESC recommends management of overweight and obese patients with HF as per guidelines for general CVD prevention [34]. The American Heart Association and the American College of Cardiology Heart Failure clinical practice guidelines highlight the lack of evidence on weight management in HF patients and accordingly it lacks specific recommendations [35].

### Limitations

There are several limitations to this study. First, we did not include ambulatory patients with HF in the analysis, so the results of this study should not be extrapolated to patients with HF who have never been hospitalized. Second, BMI was used as the only measure of obesity and we did not have data on waist circumference, percentage of body fat or other obesity metrics. Third, only 75% of patients had 1 year follow-up data and we did not have follow-up data beyond that time. The readmission rates or quality of life measures for the enrolled patients were not captured in this registry. Finally, the diagnosis of HF was made by the treating physicians at each enrolling center’s and was not validated by a core lab based on clinical and imaging data.

## Conclusions

Obesity was highly prevalent among patient admitted for decompensated HF in Egypt. Obese HF patient as compared to non-obese HF patients had higher prevalence of associated cardiometabolic disease and HFpEF phenotype on presentation. Obesity conferred a “protective effect” from in-hospital mortality, but this effect was absent at 1-year follow-up. Further studies are warranted to evaluate the effect of intentional weight loss on long-term mortality in obese HF patients.

## Data Availability

The datasets generated and/or analysed during the current study are available on request from the European Society of Cardiology/EURObservational Research Programme.

## Abbreviations

HF: Heart failure
BMI: Body mass index
EF: Ejection fraction
ESC: European Society of Cardiology
ESC-HF-LT: European Society of Cardiology Heart Failure Long-term
CKD: Chronic kidney disease
OR: Odds ratio
SBP: Systolic blood pressure
HFrEF: Heart failure with reduced ejection fraction
HFmrEF: Heart failure with mid-range ejection fraction
HFpEF: Heart failure with preserved ejection
LV: Left ventricular
DHS: Demographic and Health Survey
WHO: World Health Organization
NHP: National Hypertension Project
AF: Atrial fibrillation
OR: Odds ratio
I-PRESERVE: Irbesartan in Heart Failure with Preserved Ejection Fraction
CVD: Cardiovascular disease
CRF: Cardiorespiratory fitness
